# Survival of Patients with Multi-Level Malignant Bowel Obstruction on Total Parenteral Nutrition at Home

**DOI:** 10.3390/nu13030889

**Published:** 2021-03-10

**Authors:** Tomasz Dzierżanowski, Jacek Sobocki

**Affiliations:** 1 Laboratory of Palliative Medicine, Department of Social Medicine and Public Health, Medical University of Warsaw, 02-007 Warsaw, Poland; 2 Department of General Surgery, and Clinical Nutrition, Medical Center of Postgraduate Education, 00-416 Warsaw, Poland; jsobocki@mp.pl

**Keywords:** malignant bowel obstruction, home parenteral nutrition, malnutrition, oncology, nutrition

## Abstract

Home parenteral nutrition (HPN) may improve the survival in selected patients with malignant bowel obstruction. This retrospective, medical registry-based study aimed to identify clinical and laboratory markers predicting short survival, which would allow a more accurate selection of patients that would benefit from HPN in inoperative bowel obstruction. In a retrospective analysis of 114 patients receiving HPN, the median survival was 89 days after discharge home, and the three and six-month survival probability was 48% and 26%, respectively. Parenteral nutrition was provided during 98% of overall survival time and ended on a median of one day before the patient’s death. Discontinuing chemotherapy, anemia, severe hypoalbuminemia, and water retention appeared correlated with survival shorter than three months. In these cases, routine initiation of HPN should be discouraged, as it may not bring any benefits to the patient. The decision on the initiation of HPN should be made along with continuing or initiating chemotherapy.

## 1. Introduction

Malignant bowel obstruction (MBO) is a type of chronic intestinal failure (CIF) in which enteral nutrition cannot be provided, and the only route of administration of nutrients is parenteral. It is a frequent condition in advanced cancer, occurring in 3–15% of patients overall, with the highest incidence in ovarian (10–50%) and colorectal cancers (10–28%) [1,2,3]. MBO prognoses short life expectancy, with reported survival not exceeding a few weeks from its onset, if no parenteral nutrition is provided [2,4]. Even in otherwise healthy individuals, starvation results in death within 2–3 months [5]. The risk of death is 2.6 times higher in obstructed patients than in other CIF cases [6].

According to the European Society for Clinical Nutrition and Metabolism (ESPEN) guidelines, home parenteral nutrition (HPN) “can be considered for patients with CIF due to malignant disease” [4]. Although this recommendation reached a strong consensus (95.8% agreement), the grade of recommendation is low (0) due to the low quality of the evidence [4]. Besides, “HPN should be prescribed to prevent an earlier death from malnutrition in advanced cancer patients with CIF, if their life expectancy related to the cancer is expected to be longer than one to three months, even in those not undergoing active oncological treatment” (grade of Recommendation B) [4]. In a meta-analysis of twelve studies involving 437 patients treated with HPN due to MBO, HPN prolonged survival to a median 83 and mean 116 days, without deterioration of quality of life (QoL) [7]. In a recent Cochrane review, limited by a small number of patients included, it is uncertain whether total HPN improves survival or QoL in patients with MBO [8]. Nonetheless, its symptoms can be manageable and relief possible [2]. In some previous studies, only 30% of the patients with MBO surviving for more than three months benefited from total parenteral nutrition (TPN) [1]. Therefore, the nutritional intervention should aim to prolong life over three months or increase the quality of life.

Accurate identification of the patients that would benefit from HPN remains a challenge [4]. There is a validated nomogram to predict survival in incurable cachectic cancer patients on HPN, with Glasgow prognostic score, Karnofsky performance status, tumor site, and spread as significant prognostic factors [5]. However, no such prognostic factors have been identified for patients with MBO, and identification of patients in which benefits would outweigh potential harms that HPN brings. 

The study aimed to verify the overall survival and impact of the overall performance status, clinical symptoms, and laboratory test results at HPN initiation on patients’ survival probability with MBO. Identifying low survival markers would allow a more accurate qualification of patients to HPN that may bring on harms exceeding expected beneficial outcomes.

## 2. Materials and Methods

### 2.1. Data

Complete medical records of all consecutive patients hospitalized in 2011–2015 at the Medical Center of Postgraduate Education in Warsaw (Poland) were retrospectively screened and selected upon meeting the following inclusion criteria:

- Age > 18 years
- The diagnosis of the malignant bowel obstruction
- Total parenteral nutrition initiated in 2011–2015 and provided for at least one day at home

No exclusion criteria were applied.

Besides demographic details, the following data obtained at TPN initiation (after 2–7 days of optimizing the patient’s condition during hospitalization) were extracted:

1. 1. History of care
  - Primary cancer(s)—We classified primary cancers into five groups: colorectal, stomach, other gastrointestinal, gynecological (ovarian, uterus, cervical), and other.
  - Hospitalizations—the number of hospitalizations after TPN initiation and discharge home, regardless of the reason, including chemotherapy and interventions.
  - Ongoing chemotherapy (ChTx)—chemotherapy provided to the patient after—TPN’s initiation, disregarding the number of courses or the medications used.
  - TPN duration—the number of days after discharge home with TPN provided.
  - HPN duration—the number of days of TPN was provided at home.
  - For the analysis, we assumed the day of discharge from the hospital as day 0 of TPN and HPN—Overall survival (OS) time—the number of days between the HPN initiation date and the patient’s death.

2. Clinical assessment
  - The Eastern Cooperative Oncology Group (ECOG) Performance Status in [0–5] scale [9].
  - Body mass index (BMI).
    For the analysis, we assumed values <18 kg/m^2^ as low BMI.
  - Water retention—defined as any peripheral edema, or ascites, or hydrothorax evident in clinical examination or imaging tests.
  - Type of central venous access device (CAVD)

3. Laboratory tests
  - Hemoglobin (Hb).
  - White blood cell count (WBC)—We defined leukopenia as WBC < 4 × 10^9^/L, and leukocytosis as WBC >10 × 10^9^/L.
  - C-reactive protein (CRP).
  - Albumin.
  - Alanine transaminase (ALT).
  - Glomerular filtration rate (eGFR).

After extracting, the data were verified for their correctness by two researchers. 

The research achieved the acceptance of the Bioethical Committee of the Medical University of Warsaw, Poland, for retrospective and non-interventional surveys.

### 2.2. Statistical Analysis

In descriptive statistics, mean values and 95% confidence intervals (95 CI) were applied for normally distributed continuous variables; and medians and quartile values (Q25, Q75) for the others. Lilliefors and Shapiro–Wilk tests were used for testing normality. As all dependent variables appeared non-normally distributed, Spearman rank-order correlations and non-parametric (Kruskal–Wallis and Mann–Whitney U) tests were applied appropriately, and Pearson’s chi-squared test for contingency tables. The Kaplan–Meier method and log-rank test were used when comparing survival between two groups; and the chi-square test for multiple groups. A *p* value < 0.05 was considered statistically significant with Bonferroni correction where appropriate. All statistical analyses were performed with Statistica 13 (TIBCO Software Inc. (2017). Statistica (data analysis software system), version 13).

## 3. Results

### 3.1. Patient Characteristics

Overall, 117 adult patients with MBO had TPN initiated in 2011–2015, out of which 114 were included in the analysis (Table 1); three patients’ records were incomplete. All records were full observations (from TPN initiation to decease of a patient), and therefore none was censored. 

On the TPN’s start day, most patients were in good to moderately impaired overall performance status, and only 9.7% were assessed as ECOG 3 (capable of only limited self-care, confined to bed or chair more than 50% of waking hours). None was in ECOG 4 (completely disabled; cannot carry on any self-care; totally confined to bed or chair), as it was a contraindication to starting TPN. It is important to reiterate that the performance status (ECOG) refers to the TPN’s initiation day (day of discharge from the hospital). Patients were usually admitted to a hospital in a lower status and improved before being qualified to TPN. The initial overall performance status deteriorated after discharge over the whole period of care till death at a different pace.

### 3.2. Overall Survival

**Figure 1:**
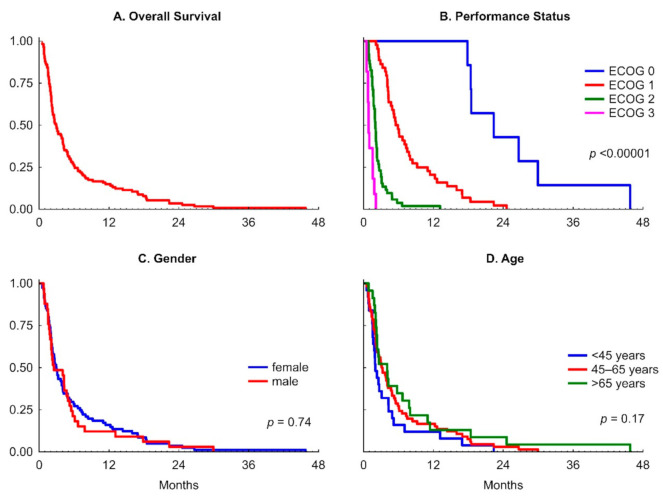
The Kaplan–Meier curves for overall survival and in subgroups: (**A**) overall survival; (**B**) performance status; (**C**) gender; (**D**) age.

As detailed in Table 2, the median overall survival time was 89 days, and the longest reached 1393 days. The OS had a right-skewed distribution with outstanding values, and the mean of 177 (95% CI 136–218 days) may be misleading and should not be considered for any comparison with external data. Therefore, we did not compare the results of other research where mean values are used as data distribution measures [10]. Three-month survival probability of 48% and the six-month of 26% were calculated (Figure 1). However, the proportion of patients with 3-month survival depended on the initial overall performance status and was 100%, 85%, and 19% in patients with ECOG 0, 1, and 2, respectively. No patient with ECOG Performance Status 3 survived longer than 2.1 months (Figure 1). 

### 3.3. Parenteral Nutrition

Patients received TPN for a median of 80 days. In a patient with the longest survival in the studied group, TPN was provided for 1386 days, out of which 1270 were at home.

On average, patients received TPN during 98% of their overall survival time and ended a median of one day (range 0–47 days) before the patient’s decease and in 18 (16%) patients until death. The median of 59% of all TPN was provided at home (HPN). 

The type of CVAD had no impact on survival (log-rank test = −0.09; *p* = 0.92) or on HPN duration and HPN/OS ratio (Mann–Whitney U test).

Fifty-five (48%) patients were hospitalized during TPN (interruption of HPN), 19 patients (17%) once and 11 (10%) twice. Two patients were hospitalized the most frequently, 12 and 13 times correspondingly. The reasons for hospitalizations were mostly chemotherapy, infections, symptom management, or deterioration of overall status and tailoring of the PN admixture.

### 3.4. Factors Affecting the Survival Time

As detailed in Table 2, the overall patient’s performance status at TPN initiation determines the average overall survival time. Figure 1 presents the Kaplan–Meier survival curves for the whole group and a comparison between groups by the initial ECOG performance status, gender, and age.

While patients with the performance status ECOG 0 at TPN initiation survived close to median two years, the patients with lower performance status lived correspondingly shorter (ECOG 1—half a year, ECOG 2—two months, and ECOG 3—less than one month). The median survival in the ECOG 3 subgroup was 26 days, with TPN provided during the median of 23 days but only the median of ten days at home (HPN).

#### 3.4.1. Gender and Age

As indicated in Figure 1, there is no statistical difference in survival between men and women (*p* = 0.74) and between age subgroups (*p* = 0.17). No correlation between age and survival time has been observed (Table 3).

#### 3.4.2. Body Mass Index and Water Retention

The median body mass index (BMI) in the whole sample was 18 kg/m^2^ (range 16–24 kg/m^2^), as shown in Table 1. However, in 54% of patients, water retention was diagnosed, so the calculated BMI was significantly higher in them (median 19 kg/m^2^) than in patients without water retention (*p* < 0.0009) and should be corrected to lower values. Therefore, we tested the impact of BMI on the probability of survival in all patients and a group without water retention, with no statistical differences in the results.

We did not find a statistical difference in survival proportions between patients with low (<18 kg/m^2^) and normal BMI (*p* = 0.36), as illustrated in Figure 1, though BMI appeared negatively correlated with survival time (R = −0.30; *p* < 0.001; Table 3).

**Figure 2:**
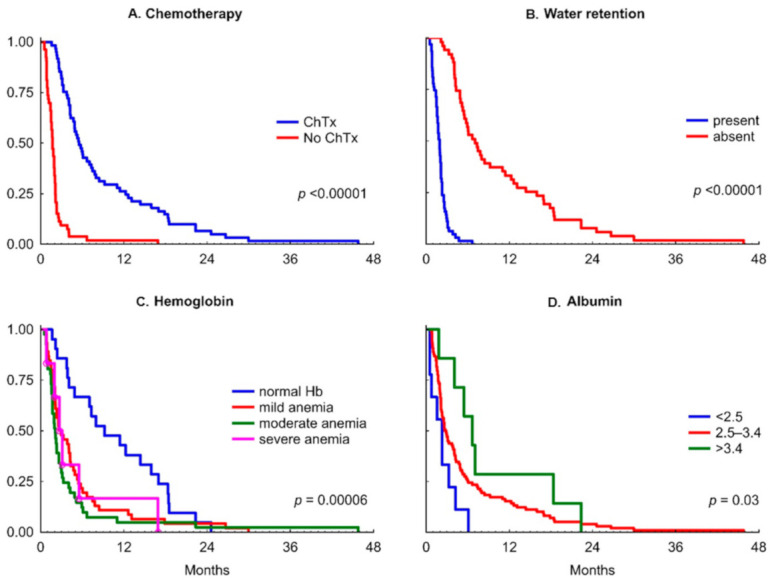
The Kaplan–Meier curves and initial clinical and laboratory values. (**A**) chemotherapy (ChTx) continued; (**B**) water retention; (**C**) hemoglobin; (**D**) serum albumin.

On the opposite, as presented in Figure 2, patients with water retention survive on TPN a median of 59 days, while significantly longer without retention—214 days, and there is a statistical difference in a log-rank test (*p* < 0.00001).

We found statistical differences in the proportion of patients with water retention dependent on ECOG Performance Status (the worse the performance status, the higher incidence of water retention; Pearson’s chi-squared test *p* < 0.00001).

#### 3.4.3. Albumin

As detailed in Table 1, the median albumin at TPN initiation was 2.85 g/dL (range 2.2–3.7 g/dL), typical for a malnourished population targeted for TPN.

The initial albumin concentration appeared well correlated with survival time (R = 0.34; *p* < 0.001); Table 3. As illustrated in Figure 2, there is statistically significant difference between the survival proportion curves between patients with different levels of albuminemia (chi-squared = 6.9; *p* = 0.03). In post hoc analysis, we found the difference between patients with normal albumin concentration (≥3.5 g/dL) and severe hypoalbuminemia (<2.5 g/dL) only (log-rank test statistic = 1.51; *p* = 0.015).

#### 3.4.4. Hemoglobin

The laboratory tests revealed a median Hb of 10.8 g/dL and that as many as 83% of patients had anemia, mostly mild to moderate (Table 1).

As shown in Figure 2, there is a difference between normal hemoglobin and anemia (*p* < 0.00006) in survival time. However, in post hoc analysis, the severity of anemia did not affect survival. In other words, any anemia resulted in significantly shorter survival of patients with MBO receiving TPN. Nevertheless, hemoglobin appeared well correlated with the survival time (R = 0.47; *p* < 0.001), to the highest degree of all independent variables (Table 3).

A positive correlation between hemoglobin and albumin concentration (R = 0.5; *p* < 0.05), and eGFR (R = 0.24; *p* < 0.05) was found.

#### 3.4.5. Glomerular Filtration Rate

The median eGFR was 69 mL/min/1.73 m^2^. However, the Spearman rank-order correlation coefficient reached 0.21 and *p* < 0.05, the result may be assumed statistically insignificant after Bonferroni correction was applied (Table 3). 

#### 3.4.6. Other Laboratory Tests

With a 95% confidence interval, mean WBC was found within the normal value range (Table 1). It appeared not correlated with survival time (Table 3). Neither leukopenia nor leukocytosis altered the survival curve (*p* = 0.48).

The mean CRP exceeded the upper norm limit of 10 mg/L, and 64% of patients had elevated CRP (Table 1). It did not correlate with survival time, nor did the elevated values impact the Kaplan-Meier survival curve. 

Alanine transaminase concentration was statistically normal in the sample. 

#### 3.4.7. Chemotherapy

As illustrated in Figure 2, in patients with MBO and TPN, the three-month survival reached 9% of patients if no chemotherapy was provided (median 58 days; Q25–Q75 31–64 days), and 82% if chemotherapy was continued (median 168 days; Q25–Q75 113–373 days); and for six-month survival, the probabilities were 2% and 44% respectively (log-rank test *p* < 0.00001). 

As expected, chemotherapy seemed to impact patients’ survival probability with MBO and TPN to the highest degree, besides water retention and overall performance status (*p* < 0.00001; Figure 2). In the post hoc analysis, for all cancer types, except “other gastroenterological” for which the sample was too small (7 cases), the differences of survival probability curves between patients with continued chemotherapy and not receiving it were statistically significant (Figure S1 in Supplementary Materials). These findings support continuing or restarting chemotherapy in MBO patients treated with HPN. 

The impact of chemotherapy on prolonging survival is undoubtful. However, despite statistical significance, the effect size could not be estimated, and causal-result relation should not be implied simplistically. It is important to reiterate that patients were qualified for continued chemotherapy, provided the overall performance status, clinical and laboratory parameters, and prognosticated lifetime met specific minimum criteria. The proportion of patients with continued chemotherapy depended on the ECOG performance status (the worse the performance status, the less the proportion of the patients with continued chemotherapy; Pearson’s chi-squared test *p* < 0.00001). Some of the independent variables of survival in this analysis, such as hemoglobin or albumin concentration values, were used to qualify patients for continuing chemotherapy. Therefore, they appeared autocorrelated with chemotherapy, as the patients with better hemoglobin or albumin were more likely to have chemotherapy continued. Consistently, in a post hoc analysis in the groups with and without chemotherapy, we found statistically significant differences for BMI (*p* = 0.015), Hb (*p* = 0.0002), albumin (*p* = 0.005), and eGFR values (*p* = 0.03) in Mann–Whitney U test, as well as for the presence of water retention (the chi-squared test *p* < 0.00001). Overall, chemotherapy’s net impact on patients’ survival with MBO receiving TPN cannot be concluded, and we were not able to perform a regression model due to the autocorrelation of the variables.

## 4. Discussion

This study was performed on full medical registry data without exclusions, which gives full information on HPN in patients with MBO in a five-year observation period. Women prevailed in the group, similarly to the epidemiological studies (59–69%) [1]. The majority of MBO patients qualified to HPN had the digestive system’s malignancy, mostly stomach or colorectal cancer. The second cause of MBO in the presented group were gynecological neoplasms, predominantly ovarian cancer. Half of the patients continued chemotherapy.

The patients qualified to HPN were mostly in a relatively good overall performance status (ECOG 0–2), and only one-tenth were ECOG 3 (confined to bed/chair >50% of time). Patients in the terminal phase of cancer with poor performance (ECOG 4) were disqualified from HPN. Although the median BMI was 18 kg/m^2^, only one-fourth of the patients had low BMI (below 18 kg/m^2^), and none was overweight. Half of them had peripheral edema, ascites, or hydrothorax, which we defined as water retention. None of the patients presented laboratory markers of renal failure.

The information on body loss before the initiation of HPN was affected by water retention or dispersed and incomplete data in the registry and did not allow for the estimation of degree of cachexia. However, the laboratory tests and clinical status revealed malnutrition in the majority of patients. Noteworthy, 94% of all patients had hypoalbuminemia, and over 80% of them—anemia. We need to reiterate that the presented clinical and laboratory values refer to the end of hospitalization before discharge, and patients had been admitted in an initially much worse condition.

### 4.1. Survival

The median survival of 89 days, 3-month, and 6-month proportions of 48% and 26%, respectively, are highly consistent with other studies [11,12]. In a meta-analysis of studies on the survival of patients with inoperable MBO and HPN, a median of 83 days was reported, with 3-month and 6-month survival proportions of 45% and 24%, respectively [7]. A recent Cochrane review reported median survival of patients with inoperable MBO on HPN between 15 and 155 days, with the range of three to 1278 days, which highly tally with our results [8]. In some studies, the survival of patients with MBO and HPN in gynecological malignancies was twice as short than in gastroenterological [7]. We did not find any significant difference. 

Likewise other studies [13], the survival depended highly on the patient’s initial performance status. While all patients with a normal overall performance status (ECOG 0) and the vast majority (85%) of those insignificantly affected by the disease (ECOG 1) lived over the next three months, only 19% of patients rated initially as ECOG 2 and none of ECOG 3 survived this long. On top of that, the median survival of patients rated as ECOG 3 appeared less than one month, and most of the last days of their life they spent in the hospital. It implies that few patients with moderately and no patients with significantly impaired performance status may benefit from HPN in terms of survival.

Gender and age did not impact survival times. In several studies, younger patients treated with TPN had better survival than older patients; however, these studies include non-malignant patients as well [14]. BMI was not associated with life expectancy. However, standardized data on the weight loss before starting TPN were unavailable. We believe that the initial body mass impact on patients’ survival with MBO and TPN should not be implied based only on the initial BMI without considering the weight loss. 

On the contrary, the clinical symptoms of water retention (peripheral edema, hydrothorax, ascites) have a high prognostic value for survival of patients nourished parenterally, with two months of expected life—if present, and seven months—if absent. As expected, water retention correlated with the severity of overall performance status.

Of the laboratory tests, the degree of anemia and albuminemia appeared best correlated with survival. Any degree of anemia appeared related to shorter survival. 

Although serum albumin correlated with survival time, the significantly lower survival trajectories were observed only when serum albumin was <2.5 g/dL. Short survival related to hypoalbuminemia was also reported in other studies, e.g., in peritoneal carcinomatosis, where albumin <2.8 g/dL was related to the survival <30 days [13]. We would like to underline that hypoalbuminemia in some patients resulted from resistant malnutrition or recurrent endotoxemia due to MBO or both.

No other laboratory tests performed before HPN, including WBC, CRP, alanine transaminase, and eGFR, appeared correlated with survival.

### 4.2. Quality of Life

Limited evidence from the systematic review suggests deterioration of QoL in highly symptomatic patients with MBO and HPN [7]. The QoL data in the registry were incomplete or assessed in different scales, so we did not decide to normalize or extrapolate them. However, we believe that health-related QoL and the patient-reported satisfaction of HPN and symptom control should be thoroughly monitored throughout all care. The QoL-adjusted days of life could be a pooled measurement of the HPN outcomes.

### 4.3. Qualification of Patients with MBO to TPN

TPN may be an option for patients with a dysfunctional intestinal tract, including MBO, to increase survival, although its impact on symptoms and QoL remains uncertain, and we did not investigate the impact of TPN on QoL either. In a recent research study, primary TPN doubled the median survival time of patients with advanced cancer cachexia, 23% of which had MBO, to 33 days vs. 15 days in patients with deficient daily calorie intake [15]. Bozetti et al. suggested that patients need to survive for longer than three months on average to gain a temporary increase in QoL [16]. Based on our study results, we suggested that HPN be administered to patients with relatively high-performance status (ECOG 0–1) and avoided in patients in worse condition. It is critical that patients were sufficiently metabolically stable to be discharged home on HPN [4]. Due to the high predictiveness of the short life expectancy of patients with MBO treated with HPN, the presence of any peripheral edema or exudation to body cavities appears discouraging against HPN initiation. Likewise, any anemia (hemoglobin <14.0 in men and <12.0 g/dL in women) severe hypoalbuminemia (<2.5 g/dL) are indicative of short survival in these patients and therefore should be taken into consideration when deciding on parenteral nutrition.

Poor patient’s performance status and improper clinical symptoms and laboratory tests, including anemia and malnutrition, are used as exclusion criteria for chemotherapy. In our study, over 80% of patients who continued chemotherapy survived three months. If chemotherapy was discontinued, patients lived a median of 58 days, and only nine percent lived three months or more. It seems that patients who do not qualify for chemotherapy may also not qualify for TPN. Therefore, the decision to initiate parenteral nutrition in MBO should be less eagerly made when discontinuing chemotherapy. This conclusion is supported by other research [5]. 

Moreover, the initiation of chemotherapy during TPN improves survival [12,17]. Likely, TPN in terminal MBO patients improves neither survival nor QoL at all, and it is chemotherapy that decides survival [11]. Our study supports previous research findings that TPN should be applied with caution and reserved for patients with a good general status before MBO, possibly effective continued chemotherapy, expected survival over three months [1]. We should reiterate that parenteral nutrition incurs additional psychosocial burden to a patient, interferes with daily activities, raises infection risk, and involves additional health system resources [18,19]. 

It is noteworthy that Naghibi et al. did not find a difference in survival curves between the groups who underwent or did not undergo chemoradiotherapy [7]. On the contrary, in our study, chemotherapy appeared correlated with the overall survival of parenterally nourished patients with MBO, and we found such a difference in colorectal, stomach, and gynecological cancer subgroups.

### 4.4. Strengths and Weaknesses of the Study

This study’s strength is that data came from a five-year registry without exclusions, which means they represent a real setting. However, like all other research, we present data only on the population treated with HPN and do not compare to the group disqualified from parenteral nutrition. Thus, we cannot tell the net difference HPN brings for the overall survival. The undoubtful weakness of this retrospective analysis is that we did not have information on QoL and precise reasons for hospitalizations (e.g., chemotherapy, infections, etc.). The data on QoL of the parenterally nourished patients remain ambiguous [8]. We believe that both survival and quality of life at its end should be measured and compared to patients untreated with HPN, which is consistent with the opinion of other researchers [20] and remains an unmet research need.

## 5. Conclusions

In light of this study, there are clinical and laboratory markers of shorter than three months survival of patients with MBO and treated with HPN. Amongst them are discontinuing chemotherapy, water retention, anemia, and severe hypoalbuminemia. In such cases, routine initiation of HPN should be discouraged. Further research on the health quality-adjusted survival should be conducted.

## Figures and Tables

**Table 1:** Patient characteristics.

Variable	Value	*n* (%)
Overall patients		114 (100%)
Gender; female		81 (71.1%)
Age at TPN initiation (years); mean (95% CI)	54.7 (52.5–56.9)	
**ECOG Performance Status**		
0		7 (6.1%)
1		44 (38.6%)
2		52 (45.6%)
3		11 (9.7%)
**Primary tumor site**		
Colorectal		19 (16.7%)
Stomach		40 (35.1%)
Other gastroenterological		7 (6.1%)
Gynecological		33 (28.9%)
Ovarian		25(21.9%)
Other gynecological		8 (7.0%)
Other		15 (13.2%)
Ongoing chemotherapy		61 (53.5%)
**Clinical assessment**		
Body mass index (BMI) (kg/m^2^); median (Q25–Q75)	18 (17–19)	
BMI low (<18 kg/m^2^)		29 (25.4%)
Water retention		63 (55.3%)
**Laboratory tests**		
CRP (mg/L); median (Q25–Q75)	15 (8–25)	
CRP elevated > 10 mg/L		73 (64.0%)
Hemoglobin (g/dL); median (Q25–Q75)	10.8 (9.2–12.1)	
Normal		21 (18.4%)
Mild anemia (male 10.0–14.0; female 10–12.0 g/dL)		46 (40.4%)
Moderate anemia (8.0–10.0 g/dL)		41 (36.0%)
Severe anemia (6.5–8.0 g/dL)		6 (5.3%)
WBC (10^9^/L); mean (95% CI)	7.75 (5.9–8.9)	
Leukopenia		6 (5%)
Leukocytosis		13 (11%)
ALT (U/L); median (Q25–Q75)	29 (22–38)	
ALT elevated >45 U/L		20 (17.5%)
Albumin (g/dL); median (Q25–Q75)	2.85 (2.7–3.1)	
≥3.5 g/dL		7 (6.1%)
2.5–3.4 g/dL		98 (86.0%)
<2.5 g/dL		9 (7.9%)
eGFR (mL/min/1.73 m^2^); median (Q25–Q75)	69 (59–79)	

ALT—alanine transaminase; CRP—c-reactive protein; ECOG—the Eastern Cooperative Oncology Group; eGFR—estimated glomerular filtration rate; HPN—home parenteral nutrition; TPN—total parenteral nutrition; WBC—white blood cells count.

**Table 2:** Survival time and total parenteral nutrition.

Variable	Median (Q25–Q75)	Min–Max
Overall survival (days)	89 (52–186)	16–1393
ECOG 0	680 (560–911)	543–1393
ECOG 1	174 (124.5–307.5)	65–748
ECOG 2	61.5 (46–81)	25–399
ECOG 3	26 (23–48)	16–64
Total parenteral nutrition (TPN) (days)	80 (47–185)	15–1386
Home parenteral nutrition (HPN) (days)	42.5 (18–132)	1–1270
% of lifetime on TPN (TPN/survival time %)	98% (96–99%)	47–100%
% of lifetime spend at home (HPN/TPN %)	59% (40–79%)	1.5–100%
TPN end (days before death)	1 (1–4)	0–47
Hospitalizations (#)	0 (0–2)	0–13

**Table 3:** Spearman rank-order correlations of survival and relative total and home parenteral nutrition times with initial clinical and laboratory parameters.

Variable	OS (days)	HPN/OS
Age	0.10	−0.11
BMI	−0.30 **	−0.09
CRP	−0.12	−0.21 *
Hb	0.47 **	0.20
WBC	−0.05	−0.20 *
ALT	−0.04	0.13
Albumin	0.34 **	0.27 *
eGFR	0.21 *	0.09

OS—overall survival; HPN—home parenteral nutrition; BMI—body mass index; CRP—c-reactive protein; Hb—hemoglobin; WBC—white blood cells count; ALT—alanine transaminase; eGFR—estimated glomerular filtration rate; * *p* < 0.05; ** *p* < 0.001.

## Data Availability

The data presented in this study are available on request from the corresponding author for any academic use upon citation of this article. The data are not publicly available due to privacy and permission restricted to publication of this article only.

## Supplementary Materials

The following are available online at https://www.mdpi.com/2072-6643/13/3/889/s1, Figure S1. The Kaplan-Meier curves presenting the impact of chemotherapy on survival in cancer clusters.
