# Supplementary material for: Survival of Patients with Multi-Level Malignant Bowel Obstruction on Total Parenteral Nutrition at Home
Source: Nutrients. 2021 Mar 10;13(3):889. doi: 10.3390/nu13030889 (PMC8000265; doi:10.3390/nu13030889)
Supplement: Supplementary file 1 [file nutrients-13-00889-s001.pdf]

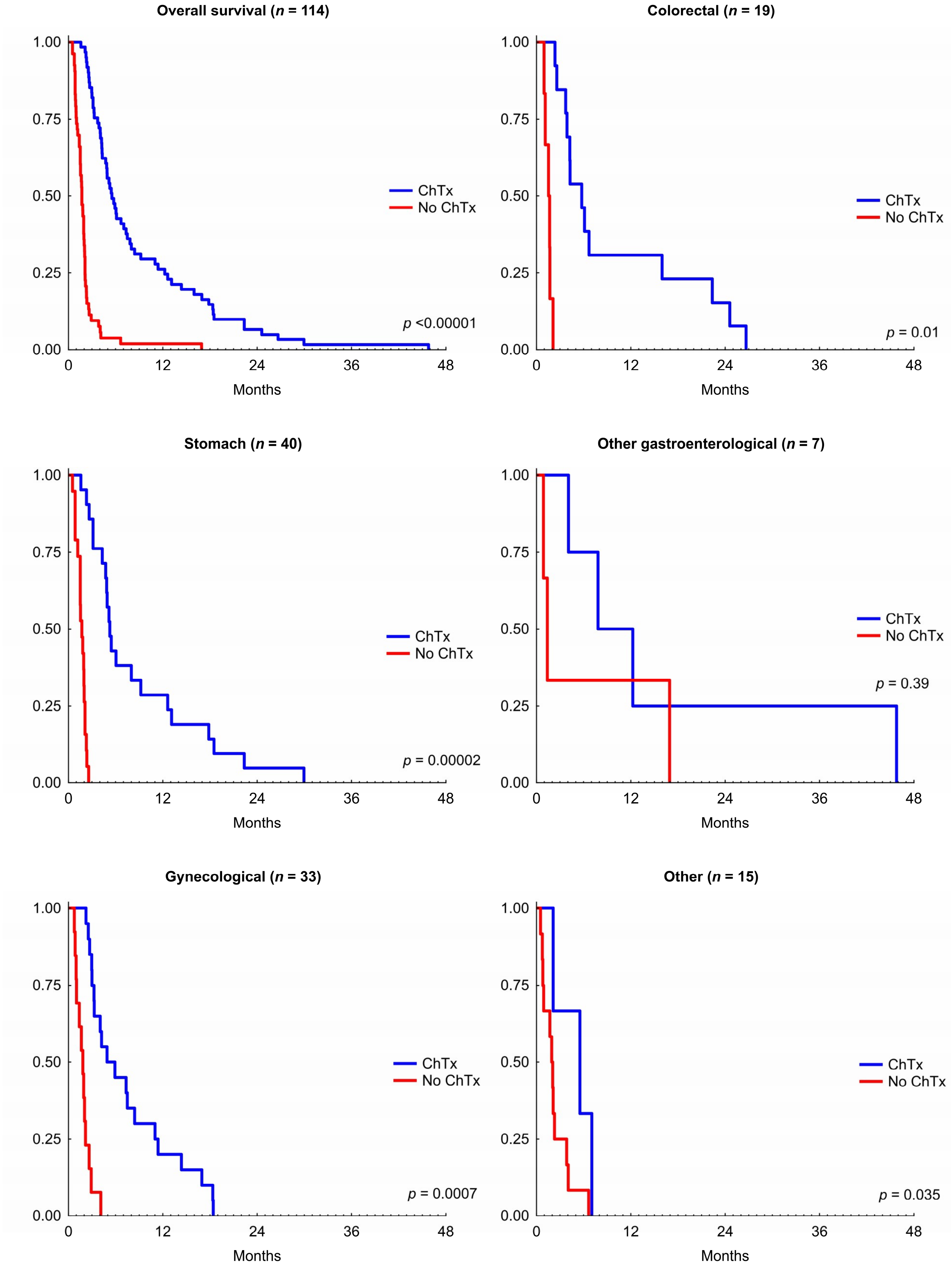

Figure S1. The Kaplan-Meier curves presenting the impact of chemotherapy (ChTx) on survival in cancer clusters.
